# Supplementary material for: Sexual signaling pattern correlates with habitat pattern in visually ornamented fishes
Source: Nat Commun. 2020 May 22;11:2561. doi: 10.1038/s41467-020-16389-0 (PMC7244530; doi:10.1038/s41467-020-16389-0)
Supplement: Supplementary file 1 — Supplementary Information [file 41467_2020_16389_MOESM1_ESM.pdf]

Supplementary Information

Sexual signaling pattern correlates with habitat pattern in visually ornamented fishes

Hulse et al.

Supplementary Notes

Supplementary Note 1: Alternative Models

To investigate random effects in our MCMCglmm models, we tested different models and compared their Deviation Information Criterion (DIC; the lower the DIC the better the model). Random effects included species and capture site nested within species (species:site). We additionally tested each model with and without the phylogenetic effect included. All models were run for 1,000,000 iterations with a burnin of 10,000 iterations and thinning interval of 50 iterations. Model results are presented in Supplementary Table 2 (males), Supplementary Table 3 (females) and Supplementary Table 4 (male vs female habitat similarity).

Supplementary Note 2: Additional Species

Our analyses initially included a population of *E. zonistium*, which was originally misidentified as the closely related and geographically adjacent *E. pyrrhogaster*[1-3]. A thorough data check by all authors prior to final revisions, including of fish and habitat images, revealed the characteristic dorsal fin stripes of *E. zonistium* in this set of images. We opted to exclude this population from final analyses to adhere to the planned experimental design of using 10 species and a relatively even representation of habitat classes. Including *E. zonistium* as an eleventh species yielded qualitatively similar results (Supplementary Figure 1, Supplementary Table 5).

Supplementary Note 3: Effect of Image Box Sizes

To ensure that our results did not depend on a particular box size, we retested our model with 400x400 and 600x600 box sizes. Since our fish images were 200x200, we upsampled them with bicubic interpolation to obtain the larger sizes. For habitat images, we sampled a larger section from the original image to achieve the larger sizes. We calculated the Fourier slope between 10 and 210 cycles per image for images with the 400x400 box size, and between 10 and 310 for the 600x600 box size. For all models, we included Species:Site as a random effect, and used the same model parameters as previously reported. These results are summarized in Supplementary Table 5 (darter-habitat correlation) and Supplementary Table 6 (male vs female habitat similarity).

Supplementary Note 4: Proportion of Images with Visible Horizon

To ensure that we were not biased in the way we photographed each habitat class, we measured the proportion of images with a visible horizon for every habitat class (Supplementary Table 7). We then tested for a difference between proportions using a two-sided proportion test in R. We found no significant difference (χ-squared: 3.7959, df = 4, p = 0.4343).

Supplementary Figures

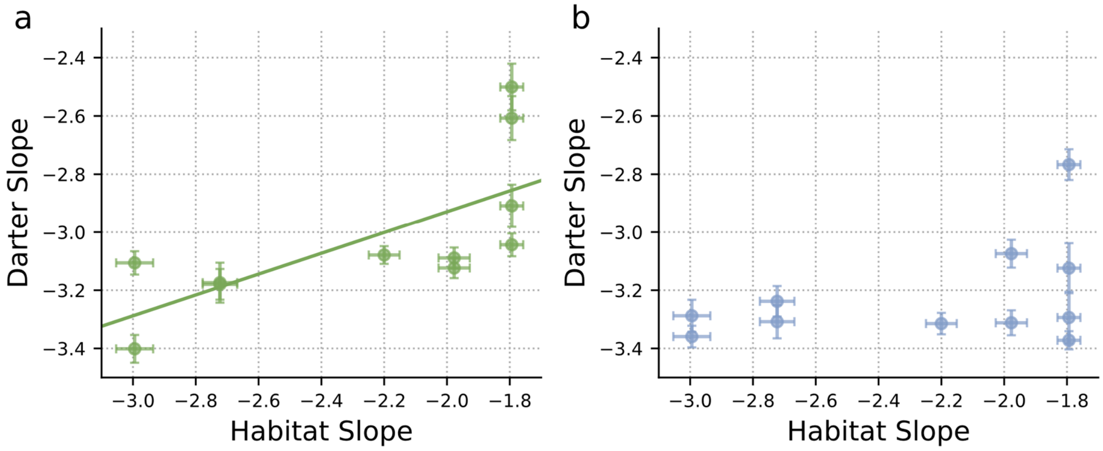

Supplementary Figure 1: Scatterplots comparing mean Fourier slopes of eleven species of *Etheostoma* and of their habitats, including *E. zonistium*. (a) Males (n = 300), (b) Females (n = 274). Error bars represent the standard error. Source data are available as a Source Data file.

Supplementary Tables

Supplementary Table 1: Locations for all field sites where darters were collected, number of individuals photographed for each species, their habitat classification, and the references used to determine the habitat classification. **E. zonistium* included in supplementary analyses (Supplementary Note 2, Supplementary Figure 1, Supplementary Table 5).

Species	Sample Sites	GPS Coordinates (E, N)	Habitat	Number Photographed (females, males)	References
E. barrenense	EF Barren River (EFBR)<br>Line Creek (LICK)<br>Trammel Creek (TRCK)	36.7459. -85.6967<br>38.6069, -85.7458<br>36.7396, -87.2896	Bedrock	18, 12<br>12, 18<br>13, 11	Etnier and Starnes, 1993[2]<br>Kuehne and Barbour, 1983[4]
E. blennioides	MF Red River (REDR)<br>Jordan Creek (JDCK)<br>Boone Creek (BNCK)	37.7815, -83.6824<br>40.3533, -87.5502<br>38.2582, -91.2832	Boulder	12, 8<br>7, 11<br>12, 11	Etnier and Starnes, 1993[2]<br>Kuehne and Barbour, 1983[4]
E. caeruleum	MF Red River (REDR)<br>Trammel Fork (TRFK)<br>Salt Fork (SAFK)	37.8149, -83.7187<br>36.7520, -86.2872<br>40.0829, -87.7806	Gravel	12, 15<br>12, 12<br>11, 11	Etnier and Starnes, 1993[2]<br>Kuehne and Barbour, 1983[4]
E. camurum	SF Kentucky River (SFKR)<br>MF Kentucky River (MFKR)<br>MF Vermillion River (MFVR)	37.3381, -83.6880<br>37.0776, -83.3926<br>40.1369, -87.7459	Boulder	12, 11<br>10, 11<br>9, 10	Etnier and Starnes, 1993[2]<br>Kuehne and Barbour, 1983[4]
E. chlorosomum	Old Town Creek (OTCK)	36.3082, -88.4488	Sand	9, 11	Etnier and Starnes, 1993[2]<br>Kuehne and Barbour, 1983[4]
E. gracile	Embarras River (EMBR)<br>Skillet Fork (UNKN)<br>Brush Creek (BRCK)	38.9074, -87.9078<br>38.7088, -88.6645<br>38.5350, -88.6121	Detritus	10, 10<br>5, 11<br>4, 12	Kuehne and Barbour, 1983[4]<br>Page & Burr, 1991[5]
E. olmstedi	M Patuxent River (PATX)<br>Rock Creek (ROCK)	39.1680, -76.8833<br>39.1510, -77.1036	Sand	11, 11<br>11, 9	Etnier and Starnes, 1993[2]<br>Kuehne and Barbour, 1983[4]
E. pyrrhogaster	Old Town Creek (OTCK)	36.3082, -88.4488	Sand	11, 11	Etnier and Starnes, 1993[2]<br>Kuehne and Barbour, 1983[4]<br>Bailey and Etnier, 1988[1]
E. swaini	Scarborough's Creek (SCRB)<br>Moaks Creek (BGCH)<br>Myers Creek (MYRC)	30.9379, -89.7782<br>31.372, -90.4395<br>31.4337, -90.42	Detritus	11, 12<br>11, 12<br>5, 11	Etnier and Starnes, 1993[2]<br>Kuehne and Barbour, 1983[4]
E. zonale	Line Creek (LNCK)<br>MF Kentucky River (MFKR)<br>Little Sugar Creek (FRCT)	36.6519. -85.8204<br>37.0776, -83.3926<br>41.5224, -80.0498	Gravel	15, 10<br>12, 10<br>7, 17	Etnier and Starnes, 1993[2]<br>Kuehne and Barbour, 1983[4]
*E. zonistium**	Barnes Fork	36.2430, -88.2874	Sand	12, 12	Bailey and Etnier[1]

Supplementary Table 2: Influence of different random effects on models predicting the Fourier slope of males by the Fourier slope of their habitat. The three tested random effects were species, site and the phylogenetic tree. The Deviance Information Criterion (DIC) indicates the quality of model fit, with the best model giving the lowest DIC. pMCMC is the Bayesian equivalent of frequentist p-values.

Species/Site Random Effects	Phylogeny	DIC	Habitat Fourier slope pMCMC
Species:Site	Yes	-22.70196	0.00657
Species + Species:Site	Yes	-22.91577	0.00727
None	Yes	115.0746	5e-05
Species:Site	No	-22.98836	0.00556
Species + Species:Site	No	-22.91459	0.00586
None	No	115.0798	5e-05

Supplementary Table 3: Influence of different random effects on models predicting the Fourier slope of females by the Fourier slope of their habitat. The three tested random effects were species, site and the phylogenetic tree. The Deviance Information Criterion (DIC) indicates the quality of model fit, with the best model giving the lowest DIC. pMCMC is the Bayesian equivalent of the frequentists' p-values.

Species/Site Random Effects	Phylogeny	DIC	Habitat Fourier slope pMCMC
Species:Site	Yes	-4.103208	0.248
Species + Species:Site	Yes	-4.134859	0.259
None	Yes	77.28863	0.0462
Species:Site	No	-4.121671	0.24
Species + Species:Site	No	-4.106409	0.248
None	No	77.28773	0.0044

Supplementary Table 4: Influence of different random effects on models predicting the deviation of the Fourier slope of fish from the Fourier slope of their habitat, by sex. The three tested random effects are species, site and the phylogenetic tree. The Deviance Information Criterion (DIC) indicates the quality of model fit, with the best model giving the lowest DIC. pMCMC is the Bayesian equivalent of the frequentists' p-values.

Species/Site Random Effects	Phylogeny	DIC	Sex pMCMC
Species:Site	Yes	57.19289	5e-05
Species + Species:Site	Yes	55.5482	5e-05
None	Yes	545.0605	0.000505
Species:Site	No	57.1904	5e-05
Species + Species:Site	No	55.51227	5e-05
None	No	545.0592	0.000505

Supplementary Table 5: Statistical results for analyses including *E. zonistium*.

Test	Mean	95% CI	pMCMC
Habitat-Darter Correspondence (Males)	0.26674	[0.09004, 0.44064]	0.00343
Habitat-Darter Correspondence (Females)	0.10714	[-0.06164, 0.2735]	0.191
Male vs. Female Habitat Matching	-0.127	[-0.159, -0.099]	5e-05

Supplementary Table 6: Influence of different image scales on models predicting the Fourier slope of fish by the Fourier slope of their habitat. All models are presented with Species:Site and phylogeny as random effects.

Sex	Image Scale	DIC	Habitat Fourier slope pMCMC
Male	400x400	-302.1904	0.00707
Female	400x400	-272.2391	0.359
Male	600x600	-427.1342	0.0119
Female	600x600	-414.9956	0.526

Supplementary Table 7: Influence of different image scales on models predicting the deviation of the Fourier slope of fish from the Fourier slope of their habitat, by sex. All models are presented with Species:Site and phylogeny as random effects.

Image Scale	DIC	Sex pMCMC
400x400	792.0467	5e-05
600x600	556.9518	5e-05

Supplementary Table 8: Proportions of habitat images with visible horizon.

Habitat Class	No. Images with Horizon	No. Images without Horizon	Proportion with Horizon
Sand	61	49	0.5545
Detritus	59	69	0.4609
Gravel	53	67	0.4417
Bedrock	52	64	0.4483
Boulder	62	66	0.4844

Supplementary References

1.Bailey, R. M. & Etnier, D. A. Comments on the subgenera of Darters (Percidae) with descriptions of two new species of *Etheostoma* (*Ulocentra*) from Southeastern United States. *Misc. Publ. Mus. Zool. Univ. Mich* **175**, 1-48 (1988).

2. Etnier, D. & Starnes, W. *The Fishes of Tennessee.* (The University of Tennessee Press, 1993).

3. Carney, D. A. & Burr, B. M. Life histories of the bandfin darter, *Etheostoma zonistium*, and the firebelly darter, *Etheostoma pyrrhogaster*, in western Kentucky. *Illinois Nat. Hist. Surv. Biol. Notes* **134**, 1–16 (1989).

4. Kuehne, R. A. & Barbour, R. W. *The American Darters*. (University Press of Kentucky, 1983).

5. Page, L. M., & Burr, B. M. *Peterson Field Guide to Freshwater Fishes of North America North of Mexico*. (Houghton Mifflin Harcourt, 2011).
