# Supplementary material for: Sexual signaling pattern correlates with habitat pattern in visually ornamented fishes
Source: Nat Commun. 2020 May 22;11:2561. doi: 10.1038/s41467-020-16389-0 (PMC7244530; doi:10.1038/s41467-020-16389-0)
Supplement: Supplementary file 3 — Reporting Summary [file 41467_2020_16389_MOESM3_ESM.pdf]

nature research

Corresponding author(s): Samuel Hulse

Last updated by author(s): April 2nd, 2020

Reporting Summary

Nature Research wishes to improve the reproducibility of the work that we publish. This form provides structure for consistency and transparency in reporting. For further information on Nature Research policies, see Authors & Referees and the Editorial Policy Checklist .

Statistics

For all statistical analyses, confirm that the following items are present in the figure legend, table legend, main text, or Methods section.

n/a	Confirmed	
☐	☒	The exact sample size (*n*) for each experimental group/condition, given as a discrete number and unit of measurement
☐	☒	A statement on whether measurements were taken from distinct samples or whether the same sample was measured repeatedly
☐	☒	The statistical test(s) used AND whether they are one- or two-sided<br>*Only common tests should be described solely by name; describe more complex techniques in the Methods section.*
☐	☒	A description of all covariates tested
☐	☒	A description of any assumptions or corrections, such as tests of normality and adjustment for multiple comparisons
☐	☒	A full description of the statistical parameters including central tendency (e.g. means) or other basic estimates (e.g. regression coefficient) AND variation (e.g. standard deviation) or associated estimates of uncertainty (e.g. confidence intervals)
☐	☒	For null hypothesis testing, the test statistic (e.g. *F*, *t*, *r*) with confidence intervals, effect sizes, degrees of freedom and *P* value noted<br>*Give P values as exact values whenever suitable.*
☐	☒	For Bayesian analysis, information on the choice of priors and Markov chain Monte Carlo settings
☐	☒	For hierarchical and complex designs, identification of the appropriate level for tests and full reporting of outcomes
☐	☒	Estimates of effect sizes (e.g. Cohen's *d*, Pearson's *r*), indicating how they were calculated

Our web collection on statistics for biologists contains articles on many of the points above.

Software and code

Policy information about availability of computer code

Data collection	To compile image slices into a single stack, we used Zerene Stacker Build T2019-10-07-1410
Data analysis	All of our Fourier Analysis was performed using custom python scripts, which are all available at https://github.com/svhulse/Fourier-Analysis. Our image processing script uses the rawpy 0.13.1 API. Statistical tests were performed in R, using the MCMCglmm 2.29 package.

For manuscripts utilizing custom algorithms or software that are central to the research but not yet described in published literature, software must be made available to editors/reviewers. We strongly encourage code deposition in a community repository (e.g. GitHub). See the Nature Research guidelines for submitting code & software for further information.

Data

Policy information about availability of data

All manuscripts must include a data availability statement. This statement should provide the following information, where applicable:

- Accession codes, unique identifiers, or web links for publicly available datasets
- A list of figures that have associated raw data
- A description of any restrictions on data availability

All data and code used to generate our figures and results are available via github at https://github.com/svhulse/Fourier-Analysis. The phylogenetic tree used is available on TreeBase at https://treebase.org/treebase-web/search/study/trees.html?id=11548. Image files are available upon request.

Field-specific reporting

Please select the one below that is the best fit for your research. If you are not sure, read the appropriate sections before making your selection.

Ecological, evolutionary & environmental sciences study design

All studies must disclose on these points even when the disclosure is negative.

Study description	We investigated whether the slope of the Fourier power spectrum in darters correlates to that of their habitats, in order to test our hypothesis that the visual statistics of the environment drive female preferences for male patterns in a sensory drive like manner. To do so, we collected approximately 30 males of 30 females for 10 species of darters, and used digital photography to analyze the visual statistics of darters and their habitats. We included the capture site for each darter as a random effect. In total, we captured and photographed 550 individuals.
Research sample	We captured approximately 30 males and 30 females for 10 species of darters (Etheostoma caeruleum, Etheostoma barrenense, Etheostoma camurum, Etheostoma blennioides, Etheostoma chlorosomum, Etheostoma pyrrhogaster, Etheostoma swaini, Etheostoma zonale, Etheostoma gracile and Etheostoma olmstedi), with a total of 550 individuals captured and photographed. These species were chosen based on their broad phylogenetic distribution and diverse habitat preferences. We believe the sample sites selected provide for a representative sample for each species. While we made sure that all individuals caught were adults, we were unable to age them beyond that.
Sampling strategy	We used a power analysis to determine the appropriate sample sizes for our study.
Data collection	Adult darters were captured in small to medium streams using kick-seining. Samuel Hulse was present for all data collection with occasional assistance from Tamra Mendelson, Julien Renoult and Natalie Roberts. They were then brought back to our indoor facility for photography. We used a DSLR with a focus stacking mount to capture highly detailed macro photography for each darter. For our habitat imagery, we used the same DSLR, in an underwater housing, and captured underwater photography detailing the areas where darters were caught.
Timing and spatial scale	Data collection took place during March-May of 2017, and 2018. During these periods, sampling occurred whenever weather, and water conditions allowed. We conducted our sampling between 10:00 and 17:00, when darters are most active. Sampling was stopped once we achieved the desired sample size for each site. Our samples were taken in the Eastern United States, across 30 sites in Maryland, Pennsylvania, Kentucky, Tennessee, Missouri, Mississippi, and Louisiana.
Data exclusions	Some of our habitat photographs were excluded from our analysis if they were out of focus, overexposed, or otherwise non-viable.
Reproducibility	We worked with common species, and used photography methods which are well described, and easy to replicate. Additionally, all of our computational methods are standard for the field, and the code we used is publicly available.
Randomization	Samples were not allocated into groups, other than by their capture site. In order to control for this, we included capture site as a random effect in all relevant models.
Blinding	Blinding was not possible for our study, as our photography methods required directly interacting with the samples.

Did the study involve field work? ☒ Yes ☐ No

Field work, collection and transport

Field conditions	We captured darters in small to medium sized streams. To ensure success, we only sampled on days where it was not raining, and water levels were sufficiently low.
Location	Our sampling ranged over 24 field sites across Maryland, Kentucky, Tennessee, Missouri, Pennsylvania, Louisianan, and Mississippi. The sites for E. barrenense were the East Fork Barren River (36.7459, -85.6967), Line Creek (38.6069, -85.7458) and Trammel Creek (36.7396, -87.2896). The sites for E. blennioides were the Middle Fork Red River (37.7815, -83.6824), Jordan Creek (40.3533, -87.5502) and Boone Creek (38.2582, -91.2832). For E. caeruleum, our sites were the Middle Fork Red River (37.8149, -83.7187), Trammel Fork (36.7520, -86.2872) and Salt Fork (40.0829, -87.7806). For E. camurum, our sites were the South Fork Kentucky River (37.3381, -83.6880), Middle Fork Kentucky River (37.0776, -83.3926) and Middle Fork Vermillion River (40.1369, -87.7459). For E. chlorosomum, our site was the Old Town Creek (36.3082, -88.4488). For E. gracile, our sites were the Embarras River (38.9074, -87.9078), Skillet Fork (38.7088, -88.6645) and Brush Creek (38.5350, -88.6121). For E. olmstedi, our sites were the Middle Patuxent River (39.1680, -76.8833) and Rock Creek (39.1510, -77.1036). For E. pyrrhogaster, our site was the Old Town Creek (36.3082, -88.4488). For E. swaini, our sites were Scarborough's Creek (30.9379, -89.7782), Moaks Creek (31.372, -90.4395) and Myers Creek (31.4337, -90.42). For E. zonale, our sites were Line Creek (36.6519, -85.8204), the Middle Fork Kentucky River (37.0776, -83.3926) and Little Sugar Creek (41.5224, -80.0498).
Access and import/export	All streams where collection took place were easily accessed via nearby roads. For all states involved, we obtained a Scientific Collection Permit. Permits for scientific collection were obtained through the Kentucky Department of Fish and Wildlife Resources (Permits SC1711119 and SC1811151), Tennessee Wildlife Resources Agency (Permits 1052 and 1424), Illinois Department of Natural Resources (Permits A17.6089 and A18.6089), Missouri Department of Natural Resources, Louisiana Department of Natural Resources (Permit SCP 183), Mississippi Department of Wildlife, Fisheries and Parks (Permit 0219184) and Maryland Department of Natural Resources (Permit # SCP201747).
Disturbance	We only included species with stable populations in our study, and kept the number of individuals captured as small as possible given our research parameters.

Reporting for specific materials, systems and methods

We require information from authors about some types of materials, experimental systems and methods used in many studies. Here, indicate whether each material, system or method listed is relevant to your study. If you are not sure if a list item applies to your research, read the appropriate section before selecting a response.

Materials & experimental systems

n/a	Involved in the study
☒	☐ Antibodies
☒	☐ Eukaryotic cell lines
☒	☐ Palaeontology
☐	☒ Animals and other organisms
☒	☐ Human research participants
☒	☐ Clinical data

Methods

n/a	Involved in the study
☒	☐ ChIP-seq
☒	☐ Flow cytometry
☒	☐ MRI-based neuroimaging

Animals and other organisms

Policy information about studies involving animals; ARRIVE guidelines recommended for reporting animal research

Laboratory animals	The study did not involve laboratory animals
Wild animals	We captured 10 species of darters (Etheostoma caeruleum, Etheostoma barrenense, Etheostoma camurum, Etheostoma blennioides, Etheostoma chlorosomum, Etheostoma pyrrhogaster, Etheostoma swaini, Etheostoma zonale, Etheostoma gracile and Etheostoma olmstedi) in the field. Darters were captured using kick-seining. We then applied stress coat to all darters and transported them in aerated tanks to our indoor facility for photography. After photography, animals were euthanized using MS-222. Euthanasia was required to be able to standardize how the darters were positioned for our photography, as well as to fully express their nuptial coloration.
Field-collected samples	Daters were housed in indoor aquaria for a maximum of three days between capture and euthanasia. All animals were kept in room temperature dechlorinated tap water from a local well, with natural photoperiods.
Ethics oversight	All animal care and use protocols were approved by the UMBC IACUC Protocol No. TM01841518.

Note that full information on the approval of the study protocol must also be provided in the manuscript.
